# Outcomes of early treatment in acute Vogt-Koyanagi-Harada disease: a 24-month retrospective analysis

**DOI:** 10.1186/s40942-026-00898-z

**Published:** 2026-08-04

**Authors:** Marcelo Mendes Lavezzo, Camillo Carneiro Gusmão, Viviane Mayumi Sakata, Fernanda Maria S. Souto, Ruy Felippe B. G. Missaka, Priscilla Figueiredo C. da Nóbrega, Emmett T. Cunninghan, Maria Kiyoko Oyamada, Carlos Eduardo Hirata, Joyce Hisae Yamamoto

**Affiliations:** 1 Department of Ophthalmology and Laboratory of Investigation in Ophthalmology (LIM 33), Faculdade de Medicina, Hospital das Clínicas HCFMUSP, Universidade de São Paulo, St. Dr. Enéas Carvalho de Aguiar, 255, São Paulo, SP 05403-000 Brazil; 2 West Coast Medical Group, San Francisco, CA USA; 3 Department of Ophthalmology, California Pacific Medical Center, San Francisco, CA USA; 4 Department of Ophthalmology, Stanford University School of Medicine, Stanford, CA USA; 5 The Francis I Proctor Foundation, UCSF School of Medicine, San Francisco, CA USA

**Keywords:** Uveomeningoencephalitic syndrome, Uveitis, Choroiditis, Immunosuppressive agents, Treatment outcome

## Abstract

**Background:**

To evaluate structural and functional outcomes in patients with acute Vogt-Koyanagi-Harada (VKH) disease, based on the timing of systemic corticosteroid (CS) initiation and the early introduction of noncorticosteroid immunomodulatory therapy (IMT).

**Methods:**

This retrospective single-centre study included 35 consecutive patients (70 eyes) diagnosed with acute Vogt-Koyanagi-Harada disease at a tertiary center in São Paulo, Brazil. Patients received methylprednisolone pulse therapy (1,000 mg/day, 3 days) followed by gradually tapered oral prednisone (1 mg/kg/day). Patients were classified by the timing of CS initiation. The early treatment group (ETG, *n* = 24) initiated CS within 30 days of onset and comprised two subgroups: CS monotherapy (Group 1, *n* = 9) and CS plus IMT within 3 months (Group 2, *n* = 15). Those treated after 30 days because of late presentation formed the late treatment group (LTG, Group 3, *n* = 11). Follow-up was 24 months with multimodal imaging and full-field electroretinography.

**Results:**

At 24 months, 98.6% of the eyes achieved BCVA ≤ 0.3 logMAR (Snellen 20/40), with no significant differences among the groups. Compared with Groups 1 and 3, Group 2 achieved a prednisone dose of ≤ 10 mg sooner (7.3 ± 1.8 months; *p* = 0.043) and had a significantly lower cumulative dose (8,719 ± 3,067 mg; *p* = 0.02). BCVA worsening episodes occurred less frequently in Group 2 than in Group 3 (6.7% vs. 59.1%; *p* < 0.001). Compared with the ETG, the LTG presented higher rates of BCVA worsening episodes (59.1% vs. 16.7%; *p* = 0.004), anterior uveitis recurrences (45.5% vs. 8.3%; *p* = 0.02), subretinal fibrosis (45.5% vs. 8.3%; *p* = 0.02), and peripapillary atrophy (72.7% vs. 22.9%; *p* = 0.003). Adverse events occurred in 7 patients (20%): 6 related to azathioprine and 1 related to CS therapy.

**Conclusions:**

Early initiation of high-dose CS in VKH disease patients was associated with fewer relapses and structural complications. Early IMT introduction provided a CS-sparing effect, although it did not significantly improve visual or structural outcomes. Prompt CS initiation remains critical; further studies should identify patients who may benefit most from early IMT.

## Background

Vogt-Koyanagi-Harada (VKH) disease is a systemic T-cell-mediated autoimmune disorder that targets melanocyte-specific proteins [1, 2]. The disease is primarily characterized by stromal choroiditis, exudative retinal detachment, and optic disc hyperemia or swelling, followed by anterior segment involvement [1, 3–5]. It accounts for 2.5%–12.5% of uveitis cases in Brazilian referral centers [6–9]; however, its prevalence varies widely, being relatively less common in Europe [10] and in the USA [11] and more prevalent in East and Southeast Asia [12, 13]. The standard treatment of VKH disease includes early high-dose corticosteroids (CS) to provide rapid control of the early signs of inflammation and prompt recovery of visual acuity (VA) [1, 14–16]. However, inflammation becomes chronic or recurrent in almost two-thirds of patients [14, 17, 18]. The use of immunomodulatory drug therapy (IMT) reduces the risk of vision loss while imposing better control of inflammation in CS refractory/intolerant cases [1, 19]. More recently, several studies have proposed early IMT associated with high-dose CS as a step toward preventing long-term deleterious side effects of CS and avoiding chronicity [20–22].

Despite different treatment protocols, early treatment with high-dose CS is, beyond doubt, the most important aspect in determining disease prognosis [1, 23]. We have recently published partial results of two prospective cohorts of patients with acute VKH disease, followed for 24 months, both with initial high-dose CS and slow tapering; the first cohort was characterized by CS therapy with or without late IMT [14], and the second cohort was characterized by CS therapy with early initiation of IMT [24]. In both cohorts, there were no differences in inflammatory, structural, or functional outcomes. To further elucidate the potential role of early IMT in VKH disease, this study aimed to evaluate the 24-month clinical outcomes of patients followed from acute onset, according to the timing of CS initiation, with or without concomitant early IMT.

## Methods

This is a retrospective interventional cohort study based on the collection of data of 35 patients (70 eyes) diagnosed with acute VKH disease (22) followed at the Uveitis Service of the Department of Ophthalmology, Hospital das Clínicas HCFMUSP, Faculdade de Medicina, Universidade de São Paulo, São Paulo, SP, Brazil, between September 2015 and September 2024. All included patients were participants in the longitudinal VKH disease study (ClinicalTrials.gov identifiers: NCT03811366 and NCT03399175) and were followed after acute disease onset for a minimal 24-month period, with a systematic multimodal and functional evaluation and predefined treatment protocols.

Exclusion criteria included other causes of uveitis, media opacities, refractive errors > 5 diopters, and/or systemic contraindications to IMT. This study received approval from the local Institutional Ethics Committee. Written informed consent was obtained from all participants, and all study methods adhered to the tenets of the Declaration of Helsinki.

All patients were treatment-naïve and initially underwent three days of intravenous methylprednisolone pulse therapy (1000 mg/day), followed by oral prednisone (1.0 mg/kg/day) with gradual tapering over at least seven months. Patients were classified according to the timing of CS initiation: early initiation within 30 days of disease onset (*n* = 24; Groups 1 and 2) and delayed initiation after 30 days (*n* = 11; Group 3). The treatment strategy reflected the prospective protocol in place at the time of enrollment. Patients treated with CS monotherapy (Group 1) were enrolled under an earlier protocol that did not include early IMT, whereas those receiving early IMT (Group 2) were enrolled under a more recent protocol incorporating early adjunctive IMT. Group 3 comprised patients who presented to our Uveitis Service more than 30 days after symptom onset and therefore initiated CS therapy late. Within the early-CS group, nine patients received CS monotherapy (Group 1), whereas fifteen received supplemental oral azathioprine (AZA; 2 mg/kg/day, up to a maximum of 3 mg/kg/day) within three months of diagnosis, constituting early IMT (Group 2). All eleven patients with delayed CS initiation (Group 3) subsequently received azathioprine as late IMT. Six of 29 IMT-treated patients (20.7%) required switching from azathioprine to mycophenolate mofetil (MMF; 2 g/day; maximum 3 g/day) due to intolerance or refractory disease.

The protocol of the ongoing longitudinal VKH disease study included clinical and posterior segment multimodal imaging examinations, performed at baseline, 1, 3 and 6 months, and every three months thereafter. Multimodal imaging examinations included fundus color photography (TRC-50DX, Topcon, Japan), fluorescein angiography (FA), indocyanine green angiography (ICGA) and enhanced depth imaging optical coherence tomography (EDI-OCT) (Spectralis^®^ HRA + OCT, Heidelberg Engineering, Germany). Clinical data were obtained from medical records. Best-corrected visual acuity (BCVA), measured with the Snellen chart, was converted to LogMAR values. In 27 patients (77.1%), ffERG recording was performed at inclusion, 30 days, and at 6-month intervals thereafter.

**Fig. 1:**
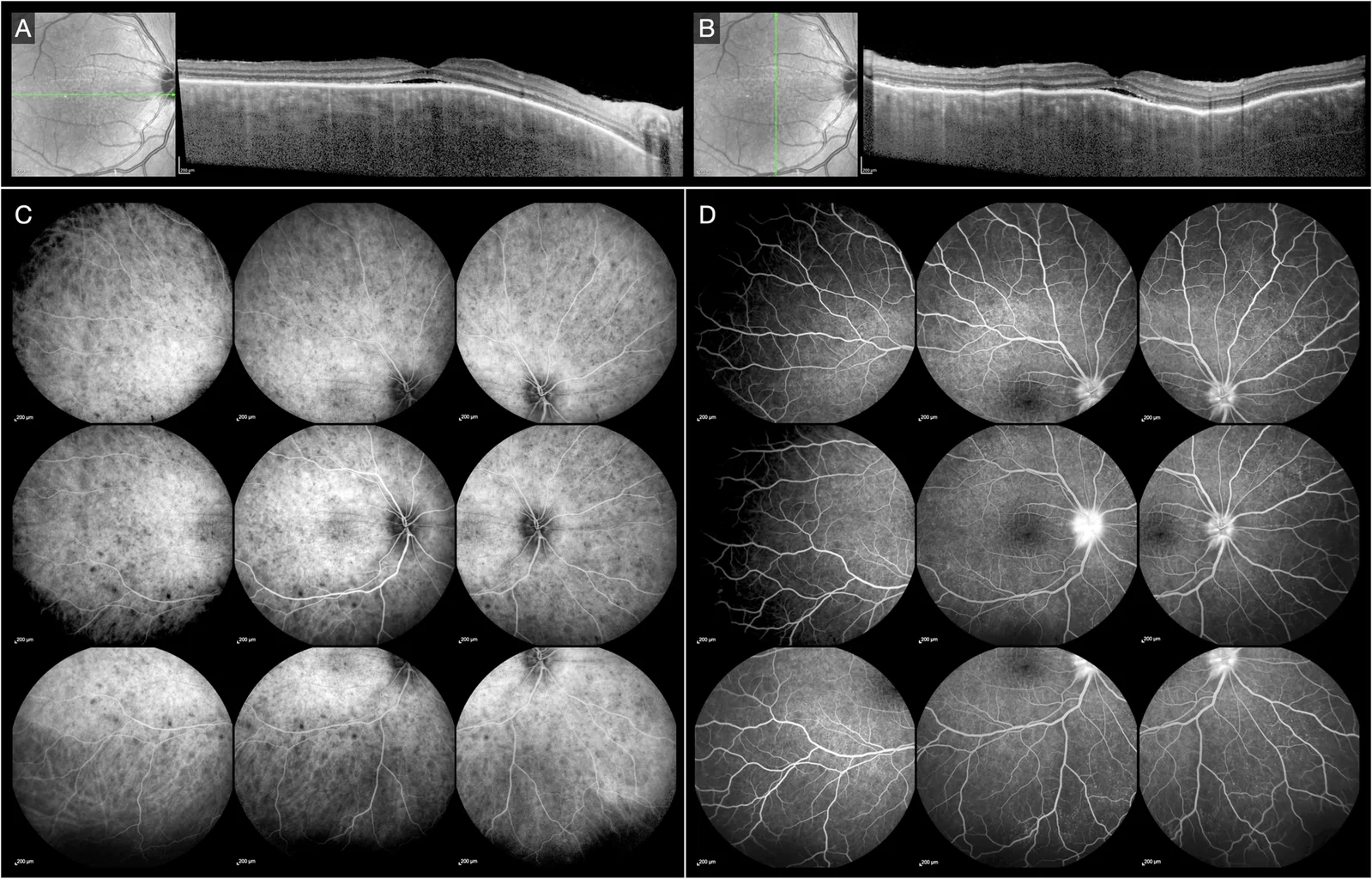
Fluorescein angiography (FA) and indocyanine green angiography (ICGA) scoring system, adapted from Tugal-Tutkun et al. (2010). (**A**, **B**) Horizontal and vertical enhanced depth imaging optical coherence tomography (EDI-OCT) scans through the fovea, providing the structural correlation required for interpretation of the angiographic findings. (**C**) ICGA montage and (**D**) FA montage, each composed of nine fields encompassing the posterior pole and peripheral quadrants, allowing systematic grading of the inflammatory angiographic signs in each region. Angiograms were assessed in both the early and late phases; the examples shown correspond to late-phase ICGA and FA montages. On ICGA (**C**), the scored signs included choroidal vasculitis *(fuzzy vessels)*, hypofluorescent dots or dark dots, and optic disc hyperfluorescence. On FA (**D**), the scored signs included optic disc hyperfluorescence, macular edema, and pinpoint leaks

**Fig. 2:**
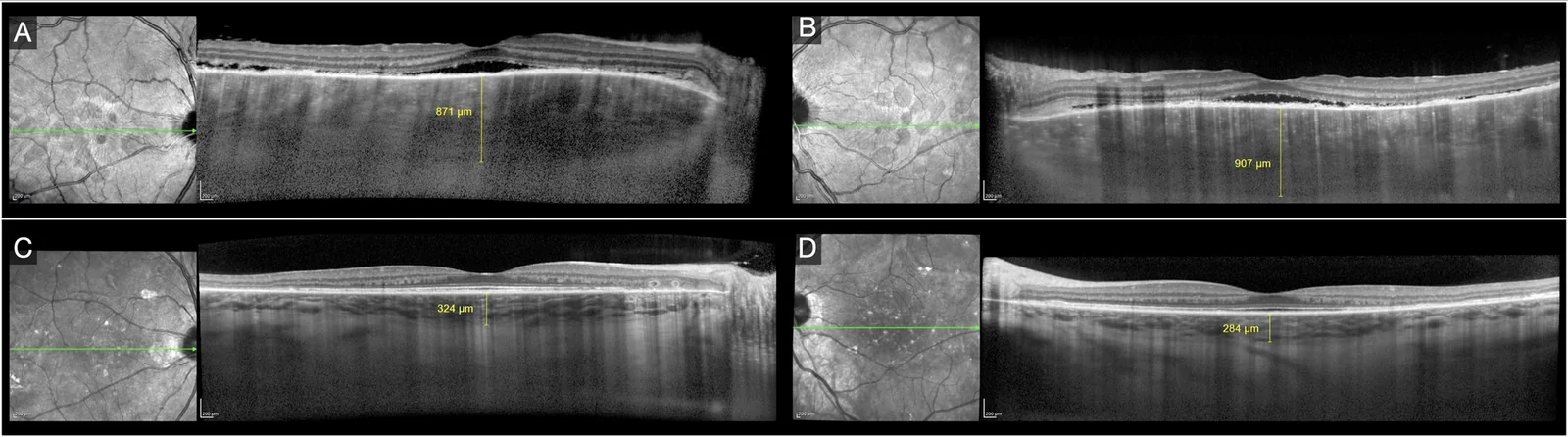
Subfoveal choroidal thickness (SFCT) measured on enhanced depth imaging horizontal optical coherence tomography (EDI-OCT) scans centered on the fovea using the Spectralis^®^ HRA + OCT (Heidelberg Engineering, Germany). In each panel, SFCT was manually measured with the built-in caliper as the vertical distance from the outer border of the retinal pigment epithelium–Bruch’s membrane complex to the choroid-sclera interface at the foveal center; the corresponding values are shown in micrometers. (**A**, **B**) Right and left eyes, respectively, of a 24-year-old female patient from Group 1 during the acute phase of Vogt-Koyanagi-Harada disease. (**C**, **D**) Corresponding scans obtained at the same locations at the 24-month follow-up

Clinical relapses after acute onset were characterized by anterior and/or posterior uveitis relapses and/or macular edema confirmed by FA, and/or OCT. FA and ICGA reading charts were adapted from Tugal-Tutkun et al., 2010 (Fig. 1) [25]. Choroidal subfoveal thickness (CST) was measured under the foveal center from retinal pigment epithelium/Bruch reflective complex to the sclerochoroidal interface (Fig. 2). During the 24-month follow-up period, the presence of ocular complications was recorded. The presence of sunset glow fundus (SGF), subretinal fibrosis (SRF) and peripapillary atrophy (PPA) was defined based on fundus color pictures. Image assessment was performed simultaneously and in consensus by three highly experienced ophthalmologists (MML, RFBGM, JHY). Choroidal neovascularization (CNV) was diagnosed with FA and/or OCT and was treated with intravitreal bevacizumab injections and IMT increment [26].

ffERG recordings were performed following the ISCEV guidelines with the RETI-port system (Roland Consult^®^; Electrophysiological Diagnostic Systems, Brandenburg, Germany) using ERG-jet electrodes (Universe SA, La Chaux-de-Fons, Switzerland) [27]. The recording sequence was scotopic rod response, scotopic cone-rod combined response, scotopic oscillatory potentials, photopic cone response, and 30-Hz flicker cone response. The normal value limits for each specific ERG response were established at 95% confidence intervals. Amplitude of *a* and *b* waves of eight averaged stimulus responses for each phase were analyzed. The results were compared with a normative database, consisting of 30 healthy subjects (60 eyes) matched for age and sex. A subnormal ffERG recording was defined as the presence of at least one parameter below the 5th percentile of healthy age, gender-matched controls.

In the ongoing longitudinal VKH disease study, systemic treatment increment during the follow-up was based on clinical relapses and/or worsening of ffERG parameters. Worsening of the ffERG parameters was defined as a value reduction of ≥ 30% in any scotopic ERG parameter, which was confirmed in two consecutive examinations [14].

The primary outcomes comparing the three treatment groups were BCVA, clinical relapses after acute disease onset, and ocular complications. Secondary outcomes included CST and FA/ICGA scores during the 24-month follow-up, in addition to the corticosteroid-sparing effect (CSE). The CSE was evaluated based on the time to reach a prednisone dose of ≤ 10 mg/d while maintaining clinically inactive uveitis, the total duration of prednisone use, and the cumulative prednisone dose.

### Statistical analysis

For the descriptive data analysis, medians, means and standard deviations were calculated for numerical variables and absolute/relative frequencies were presented for qualitative variables (Table 1). Patients had both eyes included for analysis; therefore, generalized estimation equations (GEE) were used to evaluate the difference between repeated measurements using the appropriate correction for inter-eye dependency (Tables 3, 4 and 5). GEE with Poisson’s distribution and identity link function, likelihood ratio, Kruskal-Wallis test and Fisher exact test were used when different intervals to start treatment were compared. *P* values ≤ 0.05 were considered nominal as no corrections were made for multiple comparisons. Data analysis and statistical tests were conducted using SPSS20.0 (SPSS Science, Chicago, IL, USA).

**Table 1:** Demographics and clinical data of 35 patients with Vogt-Koyanagi-Harada disease included in the study

Description		Group 1 (*n* = 9 patients, 18 eyes)	Group 2 (*n* = 15 patients, 30 eyes)	Group 3 (*n* = 11 patients, 22 eyes)	*p* value
Female *n* (%)		7 (77.8)	15 (100)	10 (90.9)	0.120^a^
Age (years)	mean ± SD	36.1 ± 13.3	33.7 ± 8.9	27.8 ± 8.6	0.174^b^
	median (range)	33 (21–67)	35 (8–30)	26 (15–43)	
Race, patients *n* (%)					
White		5 (55.6)	9 (60)	8 (72.7)	-
Mixed race		3 (33.3)	6 (40)	1 (9.1)	-
Black		0	0	2 (18.2)	-
Asian		1 (11.1)	0	0	-
HLA-DRB1*0405^c^ patients *n* (%)		2 of 6 (33.3)	2 of 11 (18.2)	4 of 7 (57.1)	0.232^a^
Pleocytosis^d^ (cells)	mean ± SD	166.2 ± 208.1	145.6 ± 151.7	147.5 ± 103.9	0.877^e^
	median (range)	76 (3-590)	113 (1-520)	200 (3-280)	

^a^ Likelihood ratio

^b^ Student’s t-test

^c^ HLA-DRB1*0405: data available of 24 patients

^d^ Pleocytosis: 2 patients missing data

^e^ Mann-Whitney test

## Results

Thirty-two of the 35 patients (91.4%) included in the study were female. The median age was 32 years (range: 15–67 years; mean ± SD: 32.5 ± 10.4) and most participants self-identified their race as White (62.8%) or Mixed races (28.6%). Among the 24 patients tested, eight (33.3%) carried the HLA-DRB1*0405 allele. Table 1 summarizes the demographic and clinical characteristics of the patients. All eyes presented serous retinal detachment (SRD) at baseline. As per group definition, the interval from symptom onset to CS initiation was significantly shorter in Group 1 (median: 11 days, range: 3–23; mean ± SD: 12.3 ± 6.7) and Group 2 (16 days, range: 8–30; mean ± SD: 16.9 ± 7) than in Group 3 (43 days, range: 32–57; mean ± SD: 42.8 ± 7.8; *p* < 0.001).

All treatment groups showed a rapid response, with marked improvement in BCVA by month 1 (Table 2). At 24 months, 98.6% of the eyes (69 out of 70) achieved a BCVA of 0.3 LogMAR or better (Snellen equivalent 20/40). Nevertheless, subnormal ffERG findings persisted in 42 out of 54 (78%) eyes. There were no between-group differences in BCVA at baseline or at any follow-up time point. Time to SRD resolution (median: 42 days, range 3–168; mean ± SD: 49.2 ± 36) and resolution of anterior chamber inflammation (median: 27 days, range: 4–105; mean ± SD: 30.9 ± 26.1) were also similar across groups. Patients in Group 2 (early IMT plus CS) achieved a prednisone dose of 10 mg sooner (median: 7 months, range: 4–11; mean ± SD: 7.3 ± 1.8; *p* = 0.043) and required a shorter overall treatment duration (median: 10 months, range: 7–15; mean ± SD: 10.7 ± 2.5; *p* = 0.047) compared to Groups 1 and 3 (Table 2).

**Table 2:** Clinical characteristics of 70 eyes with Vogt-Koyanagi-Harada disease during the 24-month follow-up, considering the start of systemic corticosteroid, associated or not with non-steroidal immunosuppressive therapy

Description		Total (*n* = 35 patients, 70 eyes)	Group 1 (*n* = 9 patients, 18 eyes)	Group 2 (*n* = 15 patients, 30 eyes)	Group 3 (*n* = 11 patients, 22 eyes)	*p* value
Best corrected visual acuity, LogMAR						
M0	mean ± SD	0.98 ± 0.78	1.06 ± 0.69	0.88 ± 0.83	1.04 ± 0.8	0.783^a^
	median (range)	0.70(0–2)	0.75 (0–2)	0.55 (0–2)	0.81 (0–2)	
M1	mean ± SD	0.21 ± 0.36	0.18 ± 0.24	0.16 ± 0.37	0.3 ± 0.43	0.437^a^
	median (range)	0.12 (0–2)	0.12 (0–1)	0 (0–2)	0.2 (0–2)	
M6	mean ± SD	0.05 ± 0.15	0.06 ± 0.15	0.02 ± 0.06	0.08 ± 0.22	0.671^a^
	median (range)	0 (0–1)	0 (0-0.6)	0 (0-0.2)	0 (0–1)	
M12	mean ± SD	0.03 ± 0.11	0.04 ± 0.13	0 ± 0.02	0.04 ± 0.15	0.891^a^
	median (range)	0 (0-0.7)	0 (0-0.4)	0 (0-0.14)	0 (0-0.7)	
M24	mean ± SD	0.03 ± 0.07	0.04 ± 0.08	0 ± 0.02	0.05 ± 0.1	0.674^a^
	median (range)	0 (0-0.4)	0 (0-0.3)	0 (0-0.15)	0 (0-0.4)	
Interval between symptoms and treatment (days)	mean ± SD	23.9 ± 14.9	12.3 ± 6.7	16.9 ± 7	42.8 ± 7.8	< 0.001^b^
	median (range)	21 (3–57)	11 (3–23)	16 (8–30)	43 (32–57)	
Interval between CS therapy and IMT introduction (days)	mean ± SD	86.5 ± 181.3	NA	22.4 ± 21.7	184 ± 294.5	0.049^c^
	median (range)	20 (0-690)	NA	20 (0–73)	28 (0-690)	
Interval to reach 10 mg of prednisone (months)	mean ± SD	10.2 ± 6.3	12.2 ± 6.6	7.3 ± 1.8	12.5 ± 8.5	0.043^c^
	median (range)	8 (4–36)	9 (4–25)	7 (4–11)	12 (6–36)	
Total time of prednisone use (months)	mean ± SD	15.4 ± 9.0	20.2 ± 8.9	10.7 ± 2.5	17.9 ± 11.8	0.047^c^
	median (range)	12 (7–42)	22 (7–35)	10 (7–15)	15 (8–42)	
Cumulative dose of prednisone (mg)	mean ± SD	11835.6 ± 5910.2	13769.5 ± 5639.3	8719.2 ± 3066.9	14502.9 ± 7375.2	0.015^c^
	median (range)	11110.0 (3705–29809)	13002.5 (6564–25937)	8430 (3705–14850)	12,222 (6507.5-29809)	
Interval to reach trace cells (days)	mean ± SD	30.9 26.1	23.4 ± 21.5	31.1 ± 26.9	36.6 ± 29.2	0.542^c^
	median (range)	27 (4-105)	14 (6–60)	19 (6-104)	30 (4-105)	
Interval to reach RD regression (days)^d^	mean ± SD	49.2 ± 35.9	47.1 ± 49.9	51.4 ± 32.2	47.9 ± 30.5	0.741^c^
	median (range)	42 (3-168)	42 (3-168)	42 (7-126)	32 (15–97)	
Need to increase immunosuppression, *n* patients (%)		9 (12.9)	3 (33.3)	0	5 (45.4)	0.004^e^
Subnormal full-field ERG at M24, *n* eyes (%)		42 (77.8)	13 (72.2)	17 (77.3)	12 (85.7)	0.767^f^

M = month; SD = standard deviation; RD = retinal detachment; ERG = electroretinogram

^a^ Generalized estimated equations with normal distribution and logarithmic link function; for all models, an exchangeable correlation matrix was assumed between the eyes of the same patient

^b^ Student’s t-test

^c^ Mann-Whitney test

^d^ Assessed by optical coherence tomography (OCT)

^e^ Likelihood ratio

^f^ Generalized estimated equations with binomial distribution and logit link function, supposing an exchangeable correlation matrix between eyes

The CST decreased in all eyes from baseline (median: 797.5 μm, range: 277–1167; mean ± SD: 780.7 ± 198.5) to M1 (median: 524 μm, range: 303–940; mean ± SD: 530.9 ± 151.4) and continued to decline through the final follow-up visit at M24 (Table 3). At baseline, FA scores differed significantly, with Group 1 (median: 4, range: 0–8; mean ± SD: 4.1 ± 2.8) showing lower values than Group 2 (median: 8.5, range: 3–10; mean ± SD: 7.6 ± 2.1, *p* = 0.001) and Group 3 (median: 9, range: 3–13; mean ± SD: 7.4 ± 2.7, *p* = 0.006). This difference was no longer significant at 12 and 24 months. With respect to the ICGA scores, no significant difference was observed between the groups at any time point (Table 3).

**Table 3:** Subclinical Inflammatory signs of 70 eyes with Vogt-Koyanagi-Harada disease at baseline (early acute phase) and during the 24-month follow-up, considering the start of systemic corticosteroid, associated or not with non-steroidal immunosuppressive therapy

Description		Group 1 (*n* = 9 patients, 18 eyes)	Group 2 (*n* = 15 patients, 30 eyes)	Group 3 (*n* = 11 patients, 22 eyes)	*p* value^c^
Subfoveal choroidal thickness (µm)^a^					
M0^b^	mean ± SD	714.17 ± 188	807.53 ± 210.06	780.35 ± 186.53	0.493
	median (range)	720.5 (434–1050)	810.5 (454–1167)	835.5 (277–1033)	
M1^b^	mean ± SD	562 ± 157.73	499.83 ± 135.69	547.73 ± 165.12	0.538
	median (range)	533 (303–911)	505.5 (310–791)	547.5 (304–940)	
M6	mean ± SD	399.33 ± 145.47	394.5 ± 104.8	436.05 ± 155.78	0.693
	median (range)	350 (244–797)	387 (208–695)	452 (175–713)	
M12	mean ± SD	409.22 ± 127.98	337.27 ± 95.01	366.36 ± 142.2	0.338
	median (range)	378.5 (284–762)	331.5 (122–693)	341 (146–649)	
M24	mean ± SD	397.28 ± 111.12	319.9 ± 90.75	332.23 ± 114.82	0.156
	median (range)	382.5 (258–635)	307.5 (139–536)	331 (114–514)	
Fluorescein angiography score^d^					
M0	mean ± SD	4.06 ± 2.79	7.57 ± 2.08	7.36 ± 2.75	0.001
	median (range)	4 (0–8)	8.5 (3–10)	9 (3–13)	
M1	mean ± SD	1.33 ± 1.32	2.63 ± 1.83	4.14 ± 2.98	0.013
	median (range)	1.5 (0–3)	2.5 (0–6)	4 (0–13)	
M6	mean ± SD	0.67 ± 0.84	2.33 ± 2.29	2.55 ± 1.92	0.049
	median (range)	0 (0–2)	2 (0–7)	2 (0–5)	
M12	mean ± SD	0.78 ± 0.81	2.97 ± 3.47	2.5 ± 1.68	0.102
	median (range)	1 (0–2)	2 (0–14)	2 (0–5)	
M24	mean ± SD	0.44 ± 0.61	0.7 ± 0.95	0.82 ± 0.73	0.536
	median (range)	0 (0–2)	0 (0–3)	1 (0–2)	
Indocyanine green angiography score^c^					
M0	mean ± SD	10.65 ± 1.88	11.56 ± 2.79	11.37 ± 3.74	0.751
	median (range)	11 (7–14)	11 (8–17)	12 (5–18)	
M1	mean ± SD	10.8 ± 1.29	9.08 ± 2.73	9.57 ± 2.79	0.237
	median (range)	11 (6.5–12)	9 (5–14)	10 (5–14)	
M6	mean ± SD	9.04 ± 1.29	8.18 ± 2.56	8.45 ± 2.52	0.668
	median (range)	9 (7–11)	7.5 (5–14)	8 (5–13)	
M12	mean ± SD	9.36 ± 1.09	7.33 ± 2.18	8.13 ± 2.3	0.052
	median (range)	9 (7.5–11)	7 (4.5–12)	8 (5–12)	
M24	mean ± SD	5.03 ± 0.81	4.7 ± 0.58	5.34 ± 1.18	0.147
	median (range)	5 (3.5-7)	5 (3–5)	5 (3.5-8)	

M = month; SD = standard deviation

^a^ Measured using enhanced depth imaging (EDI) spectral domain optical coherence tomography (SD-OCT)

^b^ Due to technical issues (e.g. considerable retinal detachment not fitting into scan), some measures of subfoveal choroidal thickness were greater than the presented measure

^c^ Generalized estimated equations with normal distribution and identity link function

^d^ Score based on Tugal-Tutkun et al. (2010)

Episodes of BCVA worsening, clinical relapses, and the frequency of ocular complications were compared among the three groups, as well as between the ETG and the LTG (Table 4). The LTG experienced a significantly higher frequency of BCVA worsening episodes (59.1% vs. 16.7%; *p* = 0.004) and of recurrent anterior uveitis (AU) episodes (45.5% vs. 8.3%; *p* = 0.02). Overall, 20% of the eyes developed cataract and 42.8% experienced ocular hypertension or glaucoma, with no significant difference between groups. Although CNV was an infrequent complication, SRF occurred at M24 in 45.5% of the eyes in the LTG compared with 8.3% in the ETG (*p* = 0.02). PPA was also significantly more prevalent in the LTG (72.7% vs. 22.9%; *p* = 0.003).

**Table 4:** Visual acuity worsening episodes, anterior uveitis relapses and ocular complications of 70 eyes with Vogt-Koyanagi-Harada disease during the 24-month follow-up according to the treatment groups

Complications, *n* eyes (%)	Early CS group			Late CS group	Total	*p* value^a^	
	Groups 1 + 2 (*n* = 48)	Group 1 (*n* = 18)	Group 2 (*n* = 30)	Group 3 (*n* = 22)		Early CS group x Late CS group	All 3 groups
Episodes of worsening VA during follow-up	8 (16.7)	6 (33.3)	2 (6.7)	13 (59.1)	21 (30)	**0.004**	**0.006**
Anterior uveitis relapse	4 (8.3)	2 (11.1)	2 (6.7)	10 (45.5)	14 (20)	**0.018**	0.062
Cataract	10 (20.8)	4 (22.2)	6 (20.0)	4 (18.2)	14 (20)	0.85	0.973
Ocular hypertension or glaucoma	20 (41.7)	8 (44.4)	12 (40.0)	10 (45.5)	30 (42.9)	0.834	0.956
Sunset glow fundus							
at M12	15 (31.3)	4 (22.2)	11 (36.7)	15 (68.2)	30 (42.9)	**0.042**	0.105
at M24	19 (39.6)	8 (44.4)	11 (36.7)	15 (68.2)	34 (48.6)	0.103	0.248
Choroidal neovascularization							
at M12	2 (4.2)	0 (0.0)	2 (6.7)	2 (9,1)	4 (5.7)	0.534	–
at M24	2 (4.2)	0 (0.0)	2 (6.7)	4 (18.2)	6 (8.6)	0.185	–
Subretinal fibrosis							
at M12	4 (8.3)	2 (11.1)	3 (10.0)	10 (45.5)	14 (20)	**0.018**	0.067
at M24	4 (8.3)	2 (11.1)	3 (10.0)	10 (45.5)	14 (20)	**0.018**	0.067
Peripapillary atrophy	11 (22.9)	4 (22.2)	7 (23.3)	16 (72.7)	27 (38.6)	**0.003**	**0.014**

CS = systemic corticosteroid; VA = visual acuity; M = month

Boldface indicates statistical significance (*p* < 0.05)

^a^ Generalized estimated equations with binomial distribution and logit link function, supposing an exchangeable correlation matrix between eyes

By the end of follow-up, SGF was observed in nearly half of the eyes. Although more frequent in Group 3, the three-group comparison did not reach statistical significance. By treatment timing, SGF incidence was significantly greater in the LTG at 12 months (*p* = 0.042), but this difference was no longer significant at 24 months (*p* = 0.1). All p values were derived from GEE (binomial family with logit link), specifying an exchangeable working correlation to account for clustering of eyes within patients. For BCVA worsening episodes, the difference was particularly notable between Group 2 and Group 3 (Bonferroni-adjusted pairwise comparisons, *p* < 0.001); beyond this, Groups 2 and 3 did not differ on any other endpoint.

Finally, seven patients (20%) experienced systemic treatment-related adverse events (TRAE), with no significant variation between the three groups. One patient required early discontinuation of oral CS therapy due to psychosis, switching to IMT. Another patient developed leukopenia and required a reduction in the dose of AZA. A third presented with fever of unknown origin, requiring discontinuation of immunosuppression. Four patients required switching from AZA to MMF: one due to drug eruption, another due to gastrointestinal intolerance, and two due to elevated hepatic transaminases (Table 5).

**Table 5:** Clinical characteristics of patients with systemic treatment-related adverse events

Patient No.	Sex	Age	Group	Comorbidities	Current IMT	Systemic TRAE
5	F	36	3	Hypothyroidism; dyslipidemia	AZA	Fever of unknown origin
7	F	38	1	Systemic arterial hypertension	CS monotherapy	Psychosis
16	F	39	2	Type 2 diabetes mellitus; previous cholecystectomy	AZA	Gastrointestinal intolerance
24	F	30	2		AZA	Drug eruption
28	F	43	3		AZA	White blood cell count abnormality
30	F	24	3	Systemic arterial hypertension	AZA	Elevated liver enzymes
31	F	48	2		AZA	Elevated liver enzymes

F = female; IMT = immunomodulatory drug therapy; CS = systemic corticosteroid; AZA = azathioprine; TRAE = systemic treatment-related adverse events

**Fig. 3:**
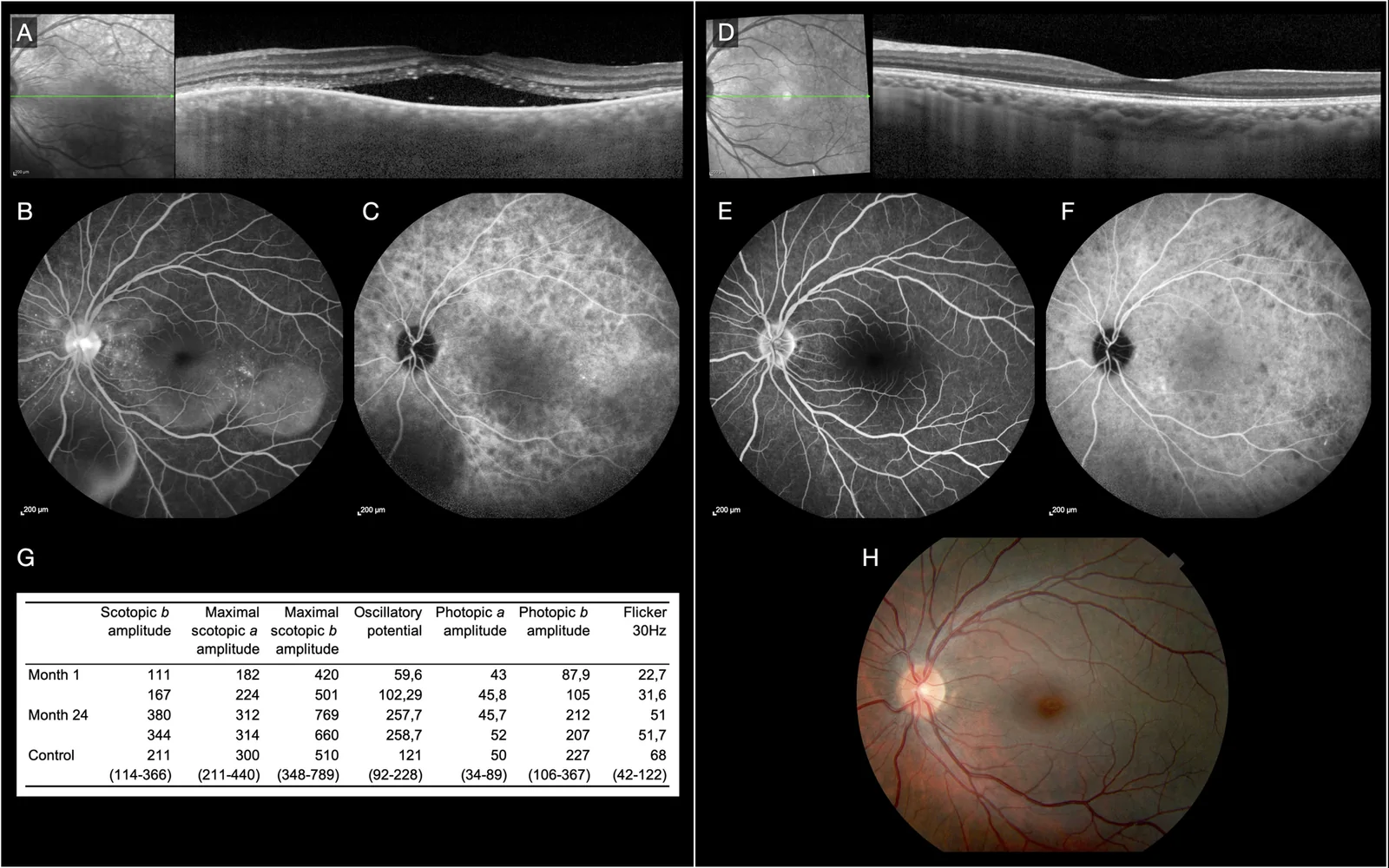
Multimodal imaging of a representative patient from Group 1 (early systemic corticosteroid therapy) at baseline and at the 24-month follow-up. *Left column (baseline*,* acute phase)*: (**A**) enhanced depth imaging optical coherence tomography (EDI-OCT) showing serous retinal detachment and marked choroidal thickening; (**B**) late-phase fluorescein angiography (FA) demonstrating multiple pinpoint hyperfluorescent leaks with pooling and optic disc hyperfluorescence; and (**C**) late-phase indocyanine green angiography (ICGA) showing numerous dark dots. *Right column (24-month follow-up)*: (**D**) EDI-OCT demonstrating complete resolution of the serous retinal detachment, with restoration of the foveal contour and normalization of choroidal thickness; (**E**) FA showing resolution of the inflammatory signs; and (**F**) ICGA showing a reduction in the number of dark dots, which nonetheless remain present. (**G**) full-field electroretinogram results obtained at month 1 and month 24 after acute disease onset. Amplitudes are expressed in µV. Data from healthy subjects (controls) are presented as median and range. (**H**) color fundus photograph obtained at month 24 showing the absence of sunset-glow fundus

**Fig. 4:**
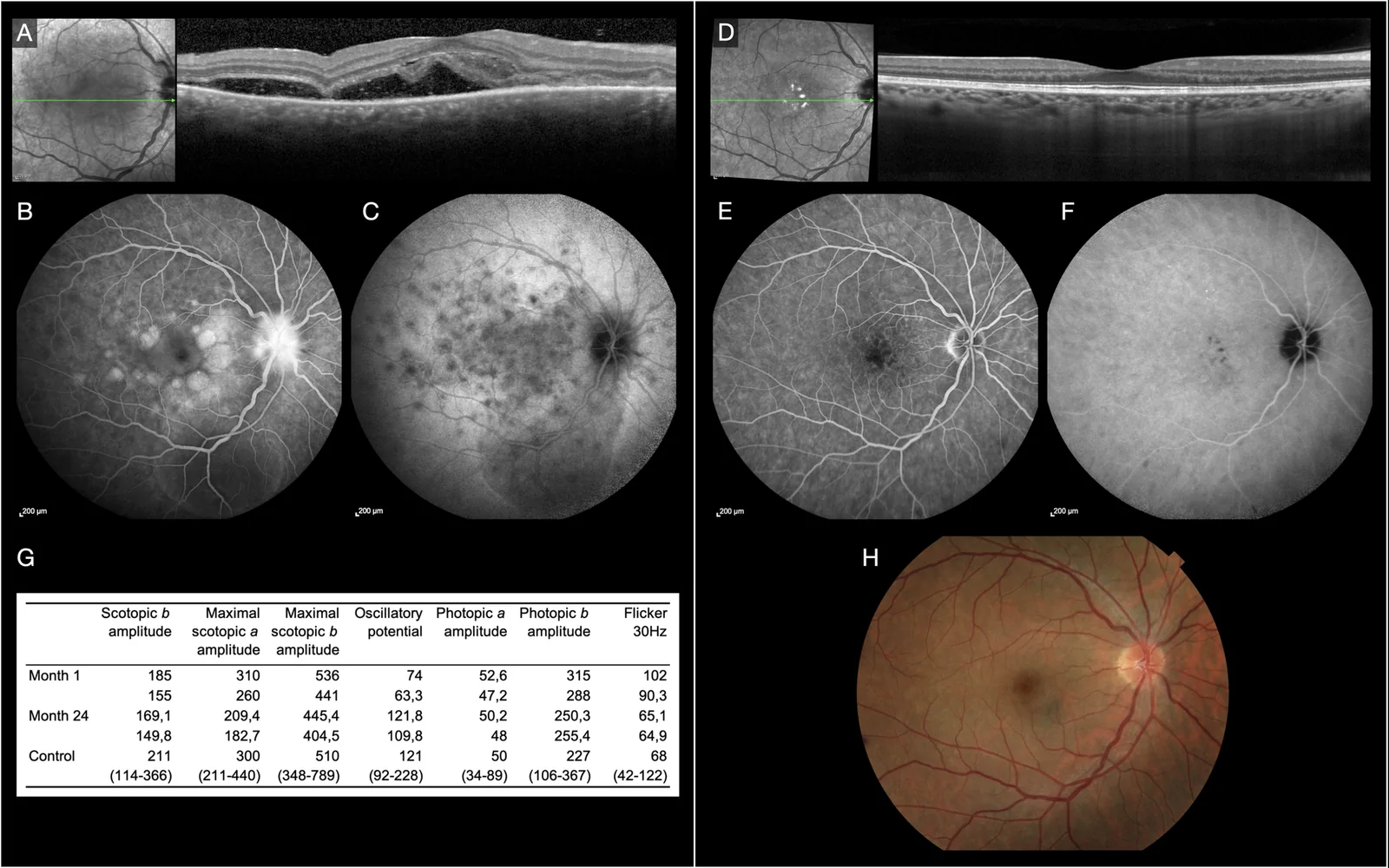
Multimodal imaging of a representative patient from Group 2 (early systemic corticosteroid therapy plus early noncorticosteroid immunomodulatory therapy) at baseline and at the 24-month follow-up. *Left column (baseline*,* acute phase)*: (**A**) enhanced depth imaging optical coherence tomography (EDI-OCT) showing serous retinal detachment with septa and choroidal thickening; (**B**) late-phase fluorescein angiography (FA) demonstrating multifocal pooling with optic disc hyperfluorescence; and (**C**) late-phase indocyanine green angiography (ICGA) showing numerous dark dots and an area of blockage corresponding to the serous retinal detachment. *Right column (24-month follow-up)*: (**D**) EDI-OCT demonstrating complete resolution of the serous retinal detachment and restoration of the foveal contour and normalization of choroidal thickness; (**E**) FA showing resolution of the inflammatory signs, with a few discrete window defects; and (**F**) ICGA showing improvement of the dark dots, with focal hypofluorescent spots corresponding to chorioretinal scars. (**G**) full-field electroretinogram results at month 1 and month 24 after acute disease onset. Amplitudes are represented as µV. Data from healthy subjects (controls) are presented as median and range. (**H**) photograph obtained at month 24 showing the absence of sunset-glow fundus

**Fig. 5:**
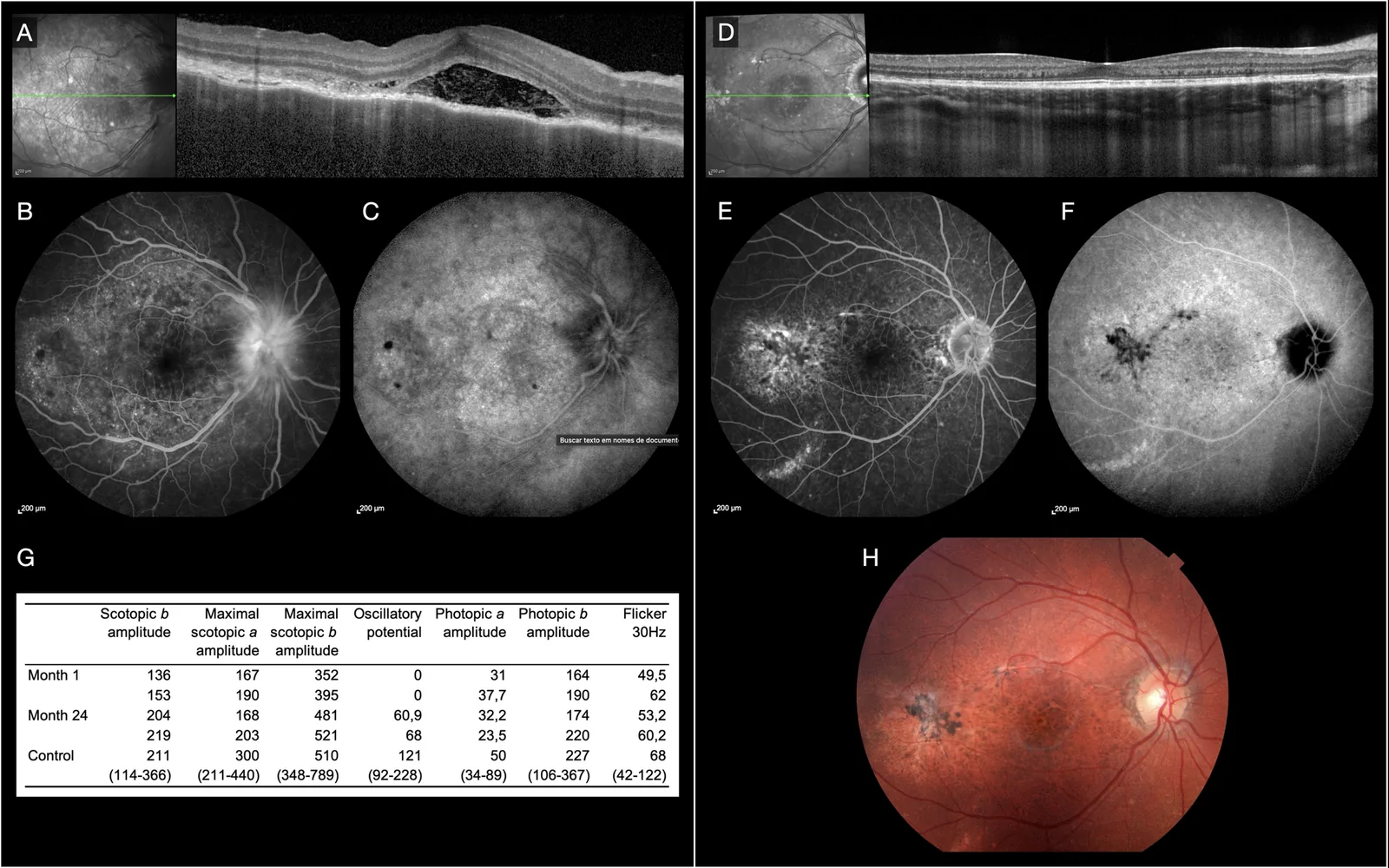
Multimodal imaging of a representative patient from Group 3 (late introduction of systemic corticosteroid therapy) at baseline and at the 24-month follow-up. *Left column (baseline*,* acute phase)*: (**A**) enhanced depth imaging optical coherence tomography (EDI-OCT) showing serous retinal detachment with retinal pigment epithelium (RPE) undulations, accompanied by choroidal thickening; (**B**) late-phase fluorescein angiography (FA) demonstrating multiple pinpoint leaks with optic disc hyperfluorescence; and (**C**) late-phase indocyanine green angiography (ICGA) showing dark dots and optic disc hyperfluorescence. *Right column (24-month follow-up)*: (**D**) EDI-OCT with resolution of the serous retinal detachment and restoration of the foveal contour, accompanied by a reduction in choroidal thickness; (**E**) FA showing areas of window defects and blockage, consistent with RPE atrophy/changes; and (**F**) ICGA showing improvement of the dark dots, with confluent hypofluorescent areas corresponding to chorioretinal scars and RPE changes. (**G**) full-field electroretinogram results at month 1 and month 24 after acute disease onset. Amplitudes are represented as µV. Data from healthy subjects (controls) are presented as median and range. (**H**) color fundus photograph at month 24 showing sunset-glow fundus with RPE mottling and peripapillary atrophy

Figures 3, 4 and 5 show a multimodal imaging analysis of representative cases of each group.

## Discussion

The optimal management of acute VKH disease has been the subject of ongoing debate for several decades. The lack of robust evidence from well-conducted randomized clinical trials has led to notable discrepancies in treatment protocols among experts, particularly regarding when to initiate IMT [28, 29]. A survey involving 104 specialists from the American Uveitis Society found that approximately 60% preferred to initiate early IMT alongside systemic CS for acute VKH disease, while nearly 40% opted to begin with CS alone, introducing IMT as necessary [28]. Although this data highlights the lack of a clear consensus, the preference for early IMT has been supported by a series of small-scale prospective studies reporting improved functional outcomes and lower rates of long-term complications [30, 31].

In this retrospective study, we evaluated the clinical outcomes of 35 patients with acute VKH disease over a 24-month follow-up period, with a particular emphasis on the timing of systemic CS initiation and the potential impact of early adjunctive IMT on disease progression. All patients received initial intravenous pulse of CS therapy, followed by a tapered oral regimen, with or without the addition of immunosuppressive agents.

As expected, most patients exhibited a prompt and favorable visual response to high-dose CS. At the one-month follow-up, mean BCVA improved from 1.06 to 0.18 logMAR in Group 1; from 0.88 to 0.16 logMAR in Group 2; and from 1.04 to 0.30 logMAR in Group 3. By the six-month assessment, all groups achieved a mean BCVA of 0.08 logMAR (approximately 20/25 Snellen equivalent) or better, with 57 of 70 treated eyes (81.4%) attaining 20/20 vision. These results are consistent with what has been reported in the global literature, supporting the favorable prognosis generally expected for patients treated during the acute phase of the disease [15, 32–36].

From a long-term perspective, our findings showed a significant association between delayed systemic treatment and the development of ocular complications. Patients in the LTG, defined as those who initiated systemic CS more than 30 days after the onset of initial symptoms, had a higher frequency of SRF and PPA (Table 5). Both conditions have been linked to unfavorable functional outcomes by other authors [37–40]. Notably, nearly half of the patients in the LTG developed SRF, with all cases presenting during the first year of follow-up. Zhao and colleagues evaluated 20 eyes from 10 patients with SRF, observing that this complication occurred within the first 4 months in all cases [41]. Whereas some authors attribute SRF to a longer, recurrent disease course [42], others have linked it to more severe initial inflammation [43].

Patients in the LTG also exhibited a higher frequency of episodes of BCVA worsening and AU recurrences (Table 5). Overall, a delay in initiating systemic CS therapy has been consistently reported as a prognostic factor for progression to chronic disease in several studies [32, 35, 44]. Some authors suggest that early treatment may halt the immune response against melanocyte proteins before the effective activation of the adaptive immune response. This could alter the natural course of the disease and prevent its progression to chronic inflammatory states [32, 45].

Regarding early non-corticosteroid IMT in noninfectious uveitis, it is widely accepted that its primary goal is to minimize cumulative exposure to systemic CS, thereby reducing the well-documented, dose-dependent adverse effects related to their use [46, 47]. Our study clearly demonstrated that the early introduction of IMT in combination with systemic CS therapy (Group 2) was associated with both a shorter total duration of prednisone use (*p* = 0.047) and a lower total cumulative dose (*p* = 0.015). The time to achieve CSE was significantly shorter in Group 2 (mean, 7.3 ± 1.8 months) compared to Group 1 (12.2 ± 6.6 months) and Group 3 (12.5 ± 8.5 months) (*p* = 0.043). Two prospective studies [30, 31] and several retrospective series have likewise reported a CS-sparing advantage when IMT is introduced early [36, 48, 49]. Whether early IMT offers benefits beyond CS-sparing effect remains a question.

A notable finding of our study was the absence of a clear benefit from early IMT initiation on functional or structural outcomes, except for a lower frequency of BCVA worsening episodes. Across all three groups, BCVA remained comparable at baseline and during follow-up, suggesting that different IMT initiation regimens may be equally effective in improving and maintaining visual acuity. Similar findings were reported in a large retrospective study by Urzua et al. in Chile, which observed no significant differences in visual improvement or complications among patients treated with first-line IMT, CS monotherapy, or late IMT [50]. Importantly, the authors also identified a high-risk subset—characterized by poor initial response to CS—in whom earlier IMT was associated with superior visual recovery, suggesting that benefits are not uniform across all patients. This idea was later reinforced by a recent risk-stratified analysis from Jin et al., which reported that immediate IMT improved BCVA and reduced SGF and relapses only in a high-risk subgroup, with no advantage seen in low-risk patients [29]. Furthermore, the immediate strategy was also associated with a higher frequency of systemic adverse events, suggesting that any potential visual gains from early IMT may be concentrated in more severe presentations and must be balanced against tolerability [29].

These findings, however, contrast with evidence from several other publications reporting superior visual outcomes in patients treated with first-line IMT plus systemic CS. In a prospective series involving 19 cases of acute VKH disease, El-Asrar et al. reported a significantly higher proportion of eyes achieving a final BCVA of 20/20 (74%) with this approach compared to a historical cohort treated with CS monotherapy (38%, *p* < 0.001) [30]. All 19 patients initially received methylprednisolone pulse therapy (1 g for 3 days), followed by oral prednisone (1 mg/kg/day) concurrently with MMF (2 g/day). Similarly, retrospective studies from Singapore [21] (29 patients) and South Korea [51] (38 patients) also reported improved long term visual acuity in patients who began IMT within 3 months of symptom onset. Although our study did not replicate these findings for final BCVA, we underscore potential methodological limitations. A ceiling effect at 24 months is plausible, given the low variability and predominance of favorable outcomes (only 2/70 eyes were ≥ 0.3 logMAR). Under such conditions, small between-group differences become harder to detect, increasing the probability of a type II error. Thus, our null results may reflect either a true absence of difference or insufficient sensitivity to detect small effects.

Since BCVA may not fully reflect cumulative damage in VKH disease, we also assessed SGF incidence as an additional outcome. Although SGF was more frequent in patients treated after 30 days (Group 3), no statistically significant difference was found in the three-group comparison (Table 5). However, when patients were grouped by the timing of CS initiation (ETG vs. LTG), the LTG showed a significantly higher SGF frequency at 12 months (68.2% vs. 31.3%, *p* = 0.04), but not at 24 months (68.2% vs. 39.6%, *p* = 0.1). We attribute this loss of significance at the final follow-up to the small sample size in each group. While these findings support a structural advantage from early initiation of high-dose systemic CS, they do not provide evidence of an incremental benefit specifically from early IMT. Conversely, El-Asrar reported excellent outcomes with early MMF use, successfully preventing SGF development in 100% of cases (19 patients) [30]. Similarly, in one of the few clinical trials on VKH disease, Ono et al. assessed the non-inferiority of combined CyA and oral prednisone versus methylprednisolone pulse therapy followed by prednisone monotherapy [52]. The authors find that the group receiving the early IMT combination exhibited significantly lower mean SGF scores at 6, 9, and 12 months of follow-up.

Indeed, early initiation of high-dose CS therapy is broadly accepted to be fundamental for rapidly suppressing inflammation and thereby reducing the risk of sequelae and chronicity [3, 44, 53]. On the other hand, it is also reasonable to assume that the use of IMT may contribute to greater long-term inflammatory stability. Our analysis revealed no differences in subclinical activity scores on ICGA, CST on OCT, or the prevalence of complications between the groups treated with early IMT and early CS monotherapy. Nevertheless, the combination therapy resulted in fewer episodes of visual acuity worsening (16.7% vs. 33.3%; *p* = 0.006), suggesting greater disease stability.

Another crucial aspect of therapeutic decision-making, however, is the safety profile of available options. Both CS and nonsteroidal immunosuppressants have known adverse effects, but robust studies support the overall safety of IMT in noninfectious uveitis [54–56]. In our study, only one TRAE (psychosis) was linked to systemic CS therapy (Group 1, *n* = 9). On the other hand, six patients experienced TRAEs associated with noncorticosteroid immunosuppressants (all related to AZA), requiring medication discontinuation or modification: three in Group 2 (gastrointestinal intolerance, elevated liver enzymes, and drug eruption) and three in Group 3 (fever of unknown origin, leukopenia, and elevated liver enzymes). Comparatively, other studies have reported varying incidences of adverse events with different treatment approaches. Ono et al., in a randomized trial of 70 patients in Japan, found a higher incidence of hyponatremia and headache with the early addition of cyclosporine A (CsA) in acute VKH disease patients, whereas no severe TRAEs were observed with CS monotherapy [52]. Similarly, a retrospective series of 79 patients revealed a significantly greater incidence of systemic adverse events in patients treated with early combination IMT than in those treated with CS monotherapy (47% vs. 7%; RR 7.1; 95% CI 2.5–20.4; *p* < 0.001) [29]. While the specific immunosuppressants involved in each event were not detailed in this series, CsA/tacrolimus were the most commonly used drugs (*n* = 33) in the cohort, followed by methotrexate (*n* = 11), MMF (*n* = 3), AZA (*n* = 1), and cyclophosphamide (*n* = 1).

The main limitation of the present study is its retrospective design and relatively small sample size, which may limit the statistical power to detect all differences between the groups and restrict the generalizability of our findings. Patient allocation was not randomized, also introducing a potential for selection bias. Additionally, multimodal imaging analysis revealed that Group 1 had significantly lower FA scores at baseline, month 1, and month 6, which may suggest a milder disease presentation in this group. Furthermore, the 24-month follow-up period, although longer than in many previously published reports, may be insufficient to estimate the true frequency of late-onset complications. Despite these limitations, the study has important strengths that add robustness to the results. Data were obtained from a prospective cohort that followed a standardized 24-month follow-up, including regular multimodal imaging (FA, ICGA, EDI-OCT) and ffERG at predefined intervals.

## Conclusions

In summary, in this retrospective study of acute VKH disease patients, early initiation of systemic CS therapy was associated with a lower frequency of structural complications and inflammatory recurrence compared to delayed treatment. While the early introduction of IMT demonstrated an evident corticosteroid-sparing effect, it did not show a clear and consistent benefit on structural and functional outcomes. These findings highlight the central role of prompt high-dose CS in acute VKH disease and support early IMT principally as a corticosteroid-sparing strategy. At the same time, they highlight the need for larger, randomized trials with greater statistical power to clarify which subgroups might gain additional benefit from immediate IMT.

## Data Availability

The data used and analyzed during the current study are available from the corresponding author on reasonable request.

## Abbreviations

AU: Anterior uveitis
AZA: Azathioprine
BCVA: Best-corrected visual acuity
CNV: Choroidal neovascularization
CS: Corticosteroid
CSE: Corticosteroid-sparing effect
CST: Choroidal subfoveal thickness
CsA: Cyclosporine A
EDI-OCT: Enhanced depth imaging optical coherence tomography
ETG: Early treatment group
FA: Fluorescein angiography
ffERG: Full-field electroretinography
ICGA: Indocyanine green angiography
IMT: Immunomodulatory therapy
LTG: Late treatment group
MMF: Mycophenolate mofetil
OCT: Optical coherence tomography
PPA: Peripapillary atrophy
SRD: Serous retinal detachment
SRF: Subretinal fibrosis
SGF: Sunset glow fundus
TRAE: Treatment-related adverse event
VKH: Vogt-Koyanagi-Harada
